# Investigation of CFRP Reinforcement Ratio on the Flexural Capacity and Failure Mode of Plain Concrete Prisms

**DOI:** 10.3390/ma15207248

**Published:** 2022-10-17

**Authors:** Hisham Jahangir Qureshi, Muhammad Umair Saleem, Nauman Khurram, Jawad Ahmad, Muhammad Nasir Amin, Kaffayatullah Khan, Fahid Aslam, Abdulrahman Fahad Al Fuhaid, Md Arifuzzaman

**Affiliations:** 1 Department of Civil and Environmental Engineering, College of Engineering, King Faisal University, Al-Ahsa 31982, Saudi Arabia; 2 Service Stream Limited Co., Chatswood, NSW 2067, Australia; 3 Department of Civil Engineering, University of Engineering and Technology Lahore, Lahore 54890, Pakistan; 4 Department of Civil Engineering, Swedish College of Engineering, Wah Cantt 47040, Pakistan; 5 Department of Civil Engineering, Prince Sattam Bin Abdulaziz University, Al-Kharj 11942, Saudi Arabia

**Keywords:** fiber-reinforced polymer, flexural strength, concrete, material

## Abstract

The utilization of carbon-fiber-reinforced polymer (CFRP) composites as strengthening materials for structural components has become quite famous over the last couple of decades. The present experimental study was carried out to examine the effect of varied widths of externally bonded CFRP on the debonding strain of CFRP and the failure mode of plain concrete beams. Twelve plain concrete prims measuring 100 mm × 100 mm × 500 mm were cast and tested under identical loading conditions. The twelve specimens include two control prisms, i.e., without CFRP strips, and the remaining ten prisms were reinforced with CFRP strips with widths of 10 mm, 20 mm, 30 mm, 40 mm, and 50 mm, respectively, i.e., two prisms in each group. Four-point loading flexural testing was carried out, and the resulting data are presented in the form of peak load vs. midpoint displacement, load vs. concrete strain, and load vs. CFRP strain. The peak load was directly recorded from the testing machine, while the midpoint deflection was recorded through the linear variable differential transducer (LVDT) installed at the midpoint. To measure the strain, two separate strain gauges were installed at the bottom of each concrete prism, i.e., one on the concrete surface and the other on the surface of the CFRP strip. The results of this study indicate that the debonding strain is a function of CFRP strip width and that the failure patterns of beams are significantly affected by the CFRP reinforcement ratio.

## 1. Introduction

Concrete is a widely used construction material in building infrastructure all around the world owing to its ability to be cast into shape, durability, cost-effectiveness, and high compressive strength [1,2,3]. Poor quality control during the construction process; the corrosion of steel reinforcement; design errors; natural disasters such as earthquakes, explosions, and hurricanes; and harsh environments are some major factors that degrade the durability and strength of reinforced concrete (RC) structures during their design life [2,4,5,6,7,8,9]. Demolishing and rebuilding or adding extra structural members to enhance the strength of a concrete structure is often regarded as an uneconomical and unsustainable strategy and thus requires huge investments [2,10].

For more than 30 years, engineers have used externally bonded fiber-reinforced polymers to strengthen and increase the load-carrying capacity of reinforced concrete structures, but due to limited test data and a lack of comprehensive design standards, the engineering community at large is not very familiar with the design and application of fiber-reinforced polymers (FRP) to strengthen existing infrastructures. Hence, more test data are required to study failure modes [11]. Guo et al. used three kinds of fibers, including glass fibers, basalt fibers, and steel fibers, to investigate their influences on the racking resistance of asphalt mixtures and adopted the digital image correlation (DIC) technique to measure the full-field strain and deformation. Different types of fiber are used to improve the performance of concrete. According to a study’s findings, a mixture’s Poisson ratio is unaffected by the fiber type. However, a mixture’s modulus is significantly impacted by the inclusion of fibers. After adding fibers, a reinforced mixture loses modulus and ultimate tensile strength. Additionally, the presence of fibers significantly increases a mixture’s ductility and slows the fracture process [12]. 

Currently, carbon-fiber-reinforced polymer (CFRP) plates and sheets are widely used in the construction industry for the strengthening and retrofitting of reinforced beams in flexure and shear [4,8,13,14,15]. CFRP possesses a high strength-to-weight ratio, flexibility, resistance to corrosion, good ductility, and ease of installation [8,16,17,18]. With the correct methodology and application of CFRP, flexure and shear cracks can be prevented in RC beams under flexure loading [8].

## 2. Literature Review

In the past, several studies have been carried out to investigate failure due to the loss of bond between the concrete and the FRP plate in an RC beam that is strengthened with the external bonding (EB) technique [19,20,21,22]. Several variables influence the capacity of RC beams strengthened with CFRP, which include the reinforcement ratio, the thickness, and the modulus of elasticity of the CFRP plate as well as the compressive strength of the concrete [20,23,24,25]. The strengthening of structural members to enhance their capacity and service life using FRP has been proven to be an effective technique. There are many different types of FRP and strengthening methods available, but strengthening structures via the use of externally bonded FRP has become very popular worldwide [26,27,28,29,30]. 

The application of FRP in RC structures utilizing normal and self-compacting concrete on mechanical and durability characteristics (i.e., installation, quality control, material selection, and environmental conditions) along with different design approaches that are available in various international design guidelines has been reviewed extensively in past, and it was concluded that more research is needed to investigate the maximum usable strain in externally bonded FRP material [1,11,31].

Thamrin conducted an experimental investigation to examine the effect of the reinforcement ratio on the flexural capacity of the beams and proposed a new model to compute the debonding moment. The results concluded that the ratio of tensile reinforcement, the ratio of the modulus of elasticity of concrete, the modulus of elasticity of the FRP plate, and the plate thickness have a significant influence on the debonding moment value [19]. Garcez conducted a beam test to assess the bond mechanism and the transfer of stresses at the CFRP/concrete interface and found that the mechanism of damage initiation and debonding propagation is dependent on the load transferring and stress redistribution after the concrete crack [20]. Yuan conducted experimental testing on CFRP-plate-reinforced concrete beams, and the results were discussed in terms of load deflection, failure modes, and crack propagation. He found that the postcracking stiffness and bearing capacity can be improved by adopting additional anchoring measures [32]. 

Salama tested nine reinforced concrete beams, out of which eight were strengthened in flexure with different configurations of bottom-bonded and side-bonded CFRP sheets, and the results were presented in the form of load–deflection response curves, failure modes, and ductility. Moreover, he found that the side-bonded strengthening scheme is a good alternative to the bottom-bonded scheme for strengthening RC beams in flexure, especially if the beam soffit is narrow or inaccessible for strengthening [13]. Choobbor focused his studies on the flexure behavior of reinforced concrete beams that are strengthened with hybrid carbon and basalt fiber reinforced polymer, and for that purpose, he tested 10 beams, of which 9 were strengthened with different combinations of CFRP and BFRP sheets, and he found that there was an improvement in the load-carrying capacity and ductility of the strengthened beam specimens [6]. 

Saleem et al. determined the moment–curvature response of seven reinforced concrete beams strengthened with different amounts and layouts of CFRP reinforcement. The study showed that increasing the CFRP reinforcement above certain levels does not help appreciably, and that the structural performance can be optimized through an appropriate combination of CFRP flexural and shear reinforcement [7]. Soares investigated the bond strength of an externally bonded reinforcement system with CFRP via an experimental program composed of a single-lap shear test. His research focus was to study the bond behavior between CFRP and a concrete surface, i.e., the type of concrete surface preparation method used before the application of CFRP, and the bond length [14]. Saleem investigated CFRP application and the reinforcement ratio in reinforced concrete beams, and the results were presented in terms of moment–curvature values, ultimate load, and failure patterns. It was noted that the failure modes of beams are dependent on the CFRP layout [8]. Qureshi studied the flexural and shear strains of polymer composites at the bonding interface of epoxy and concrete and found that the strength increment in flexural members depends on the values of the strains in the CFRP [4]. Mahendra conducted experimental testing on reinforced concrete beams strengthened with CFRP and GFRP with a suitable pattern of wrapping the beam, and his focus was to find the optimized technique to strengthen reinforced concrete beams in shear and flexure with the suitable pattern of wrapping the beam. His results showed that the beams that were strengthened with CFRP showed better results than those strengthened with GFRP [2]. Abid conducted an extensive review of the past research work in relation to the bond behavior, testing techniques, and model to investigate the bond strength. Moreover, flexural, shear, and fatigue behavior have been intensively reviewed [15]. Amran conducted a review of past research work in relation to the FRP design, material properties, and serviceability [33]. Yin conducted a single shear test on three different strength concrete specimens with six types of interface roughness to evaluate the roughness of the concrete interface and the bond performance between FRP and concrete. He found that a concrete interface with a roughness of 0.44 shows the best results in terms of interfacial bonding performance [27]. Saribiyik conducted experimental testing on reinforced low-strength concrete beam specimens that were strengthened with CRFP and GFRP wraps to examine the flexural strength and ductility. He found that the specimens that were retrofitted with GFRP showed low flexural and shear strengths compared to the CFRP specimens. However, the ductility and energy absorption capacity of the GFRP specimens were higher [34]. Bilotta conducted flexural testing of reinforced concrete beams that were strengthened with both NSM and EBR techniques to investigate the debonding behavior [22]. Colombi conducted push–pull experimental testing of both FRP warps and strips of different lengths to evaluate the debonding load [30]. Sen conducted comparative experimental testing on reinforced concrete beams strengthened with JFRP, CFRP, and GFRP to investigate the effect of strengthening on the ultimate load, ductility, and deflection [35]. Dong conducted experimental testing on reinforced concrete beams that were retrofitted in different strengthening arrangements in shear and flexure with CFRP and GFRP to evaluate the flexure and flexure–shear strength capacities. He found that the flexural shear strengthening arrangement was more effective than the flexural arrangement [36]. Sobuz, Obaidat, El-Ghandour, and Ahmed investigated the shear and flexural behavior of reinforced concrete beams strengthened by CFRP laminates via experimental testing [24,37,38,39].

The problem facing externally bonded reinforcement and the near-surface mounted technique is premature debonding, which is due to the interfacial shear stresses between the concrete and FRP, i.e., bond behavior [10,22,30]. The load-carrying capacity of reinforced concrete beams can be increased by attaching fiber-reinforced polymer composites to the tension side. However, due to the debonding failure of FRP at strains lower than the strain capacity of FRP, strengthening is compromised [40]. The load-carrying capacity of strengthened reinforced concrete members depends on the thickness as well as the width of CFRP, and there is a need to find the optimum length and width of CFRP strips [21,31,36]. Moreover, there is inconsistency between different design guidelines in relation to the use of maximum usable strain in externally bonded CFRP plates. Hence, there is a need to study the strain values that are transferred from CFRP to concrete, which are linked with the debonding failure mechanism [4,11].

Although the ultimate strength of over-reinforced beams was only slightly affected, the structural behavior of the beams was greatly enhanced by increasing the FRP reinforcement ratio [41]. The test findings demonstrate that adding more FRP reinforcements improves the flexural behavior of the structure, including its load-bearing capacity, postcracking stiffness, and deformation capability. However, a decrease in ductility was observed at a greater number of FRP reinforcements [42]. The experimental findings demonstrated that the failure mechanism of the specimen reinforced with CFRP was controlled by CFRP debonding, followed by concrete crushing. However, the control beam collapsed in concrete crushing after yielding the steel bars, which is a ductile failure. The CFRP layer reduces the ductility and toughness of the RCC beams while increasing their strength and initial stiffness [43]. The experimental findings demonstrated a considerable improvement in the ultimate load-bearing capability due to the CFRP sheet. The increase in flexural capacity over the strengthened control specimen varied from 28 to 102 percent [44]. High-strength concrete increased the cracking moment while decreasing the crack width and spacing. However, the FRP reinforcement ratio only impacts the crack width and spacing but does not affect the crack moment. As per Loukidis, in the last stress phases before fracture, a macrocrack network around the fracture region changes the sample resistance and could be a prefailure indicator [45]. The exposed conventional fire beams that were reinforced with CFRP, according to research [46], increased the strength more than the control beam. According to experimental findings, CFRP may significantly increase the flexural and tensile capacity. However, the effect of CFRP on the compressive capacity was negligible [47]. It can be concluded that CFRP improved flexural cracking behaviors and tensile capacity considerably but decreased ductility, particularly at a higher reinforcement ratio. Furthermore, CFRP has little or no impact on the compressive capacity of concrete.

## 3. Research Significance

In past, some researchers have investigated the bond strength of an externally bonded CFRP with a concrete surface via an experimental program composed of a single-lap shear test [14], which provides a debonding behavior in a pure shear environment. However, in this study, the debonding strains of CFRP and concrete are determined under pure flexural loading. For many years, it has been a challenge to accurately determine the amount of strain transferred from the concrete surface to the FRP. The strain transferred from the concrete to the FRP determines the effectiveness of the bond, which highly depends on the epoxy or adhesive used. Based on the above literature review, very few studies have investigated the debonding behavior and flexural shear strain transfer between concrete and FRP under pure flexural bending.

The strain transferred from concrete to FRP will determine the overall strengthening achieved based on the stiffness of the FRP used for the strengthening purposes. All the focus of FRP strengthening design codes has remained to theoretically determine the amount of strain that can be achieved for a given reinforcement ratio of FRP.

In this study, samples with different FRP ratios were tested under identical conditions, and the strain between concrete and FRP was closely monitored and presented for validation and evaluation for future studies. To investigate the pure flexural shear strain transfer from concrete to FRP, no steel reinforcement was provided inside the concrete prisms. To enable guidelines for future design recommendations and to facilitate strain evaluation model studies, the CFRP and concrete strains are presented and discussed in detail. For this, experimental testing was carried out to study the effect of varying the CFRP width on the flexural strains of the concrete beam specimens. All beam specimens were cast without internal longitudinal or shear reinforcement to investigate the pure concrete and CFRP strain transfer under a flexure load. During the flexural load test, the load, deflection, and strain values of CFRP and concrete were monitored closely.

## 4. Experimental Program

**Figure 1:**
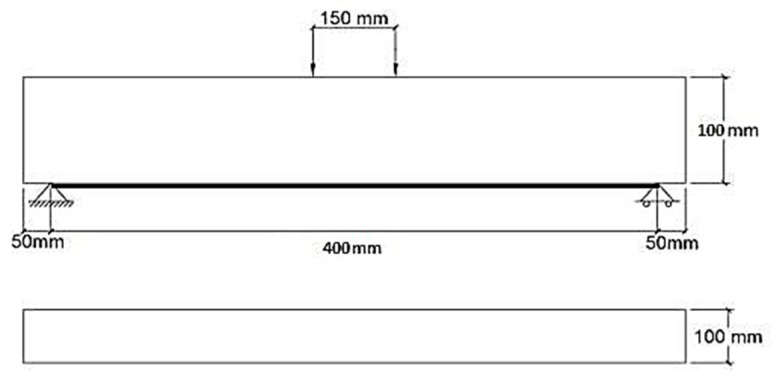
Beam dimensions.

**Figure 2:**
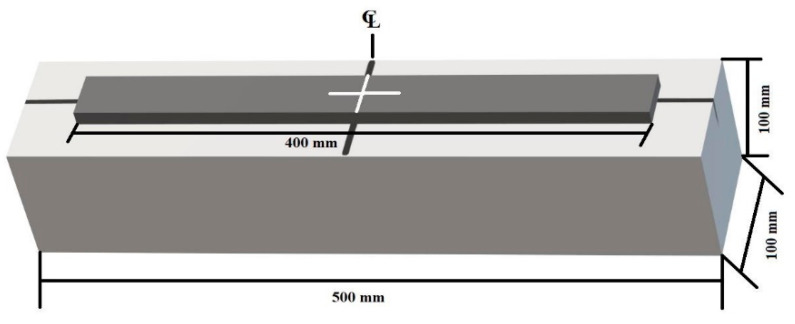
Beam assembly.

A total of twelve plain concrete prims without any internal longitudinal or shear reinforcement measuring 100 mm × 100 mm × 500 mm were prepared and tested with a clear span of 400 mm and a total length of 500 mm, as shown in Figure 1 and Figure 2.

**Figure 3:**
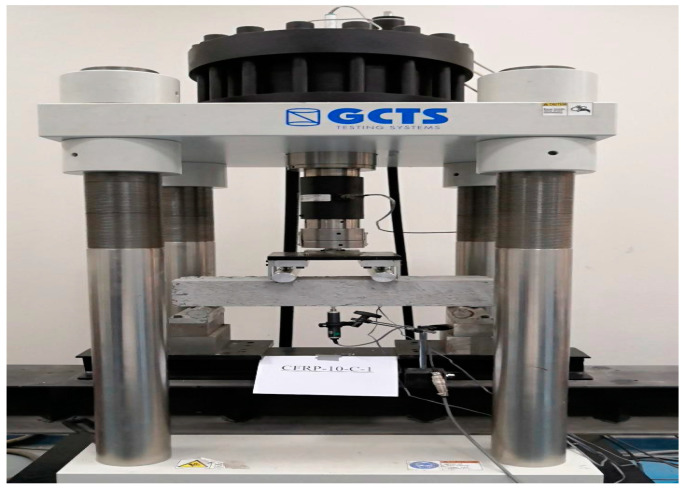
Experimental setup to test the specimen.

Four-point load tests were conducted on the beam samples. This loading arrangement is per ASTM-C1399 [48] with slight variations in the mid and end-span distances. The experimental setup of the testing specimens is shown in Figure 3.

The concentrated load was applied with a constant displacement rate of 1.5 mm/min. This displacement rate was decided based on the ASTM C1609 [49] guidelines to have the first-peak deflection between 40 to 100 s from the start of the test. Two separate strain gauges were installed on the bottom surface of each concrete prism to monitor the strain values, i.e., one on the concrete surface and the other on the surface of the CFRP strip. The proposed tests specimens without steel rebars and the loading setup elaborate the concrete and CFRP interaction under flexural stresses. This testing arrangement gives a better understanding of strengthening mechanisms under NSM (near-surface mounted) strengthening techniques where FRP is applied on extreme fibers and subjected to bending actions. A linear variable differential transformer (LVDT) was installed at the center point of the specimen to measure the vertical deflection of a simply supported concrete beam specimen.

## 5. Sample Preparation

Out of twelve specimens, two samples were prepared without CFRP strips and are referred to as control specimens, and ten samples were prepared in a group consisting of CFRP strips with widths of 10 mm, 20 mm, 30 mm, 40 mm, and 50 mm. To study the effect of varied CFRP widths on strength, deflection, and strain, a range of CFRP widths was selected that covers 10% to 50% of the surface area of the concrete on the tension face of the prisms. An evaluation of the debonding strain was one of the objectives of the study. Therefore, CFRP widths up to 50 mm with 10 mm increments were considered. It was desirable to measure the CFRP and concrete strain at the extreme fibers, with widths higher than 50 mm, as the exposed area of concrete on either side of the CFRP strip remains too narrow to measure the strain values. Debonding strain is defined as the strain at which debonding starts and the peak load starts decreasing. Once this maximum value of strain (debonding strain) is achieved, it starts losing its strength, and delamination occurs. The length and thickness of the CFRP strips in the strengthened specimens were kept constant, equal to 1.5 mm and 400 mm, respectively. The designation of all test specimens is given in Table 1.

The designations C-1 and C-2 represent control specimen 1 and 2, whereas the typical designation 10-C-1 represents specimen 1 strengthened with a CFRP strip with a 10 mm width.

**Figure 4:**
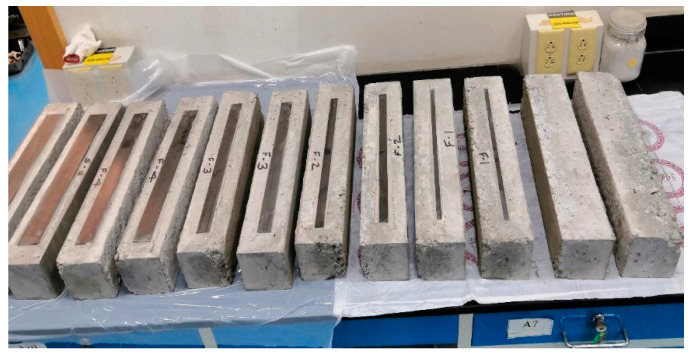
Completely prepared test specimens.

Concrete beam samples were prepared from concrete with an average compression strength of *fc′* = 32 MPa, which was determined as per ASTM C39 [50]. The specimens were the first cast in a formwork and were later cured as per ASTM C-31 [51]. After curing, the prism surfaces were cleaned with a wet cloth, dried, cleaned with compressed air, and later externally reinforced with CFRP strips. Sikadur-330 epoxy was used to attach the CFRP strips to the bottom concrete surface. CFRP strips were attached to the surface of concrete using a wet layup method and then cured for a minimum of three days after the application of CFRP. The proper quality control measures were adopted to maintain a uniform epoxy thickness over the bottom concrete beam surface. All completely prepared test specimens are presented in Figure 4.

## 6. Material Properties

Keeping in view the local construction practices, 32 MPa concrete was adopted for this study. A nominal mix ratio of 1:2:3 was adopted for 32 MPa standard concrete in which one part of Portland cement was mixed with two parts of fine dry sand and three parts of coarse aggregate. A water/cement ratio of 0.42 (air-entrained) was considered and maintained for all batches of concrete mixes. The other properties of the concrete, such as the elastic and shear moduli of elasticity, were determined using ACI 318-19 [52]. However, the material properties of the CFRP and epoxy were considered as provided by the manufacturer. The material properties of the concrete, CFRP, and epoxy are presented in Table 2, Table 3 and Table 4, respectively.

## 7. Results and Discussion

### 7.1. Peak Loads and Midspan Deflections

During experimentation, vertical displacement and the applied load were acquired using a data acquisition system. The experimental results of all specimens are summarized in terms of their peak load, midspan deflection, and their average values in Table 5.

It was observed that all beam samples that were strengthened with externally bonded CFRP depicted better results compared to control specimens (C-1 and C-2) in terms of load-carrying and deformation capacities. The beam sample strengthened with a 50 mm CFRP strip (50-C-2) showed the highest values of peak load (27.65 kN) as well as deflection (1.996 mm).

**Figure 5:**
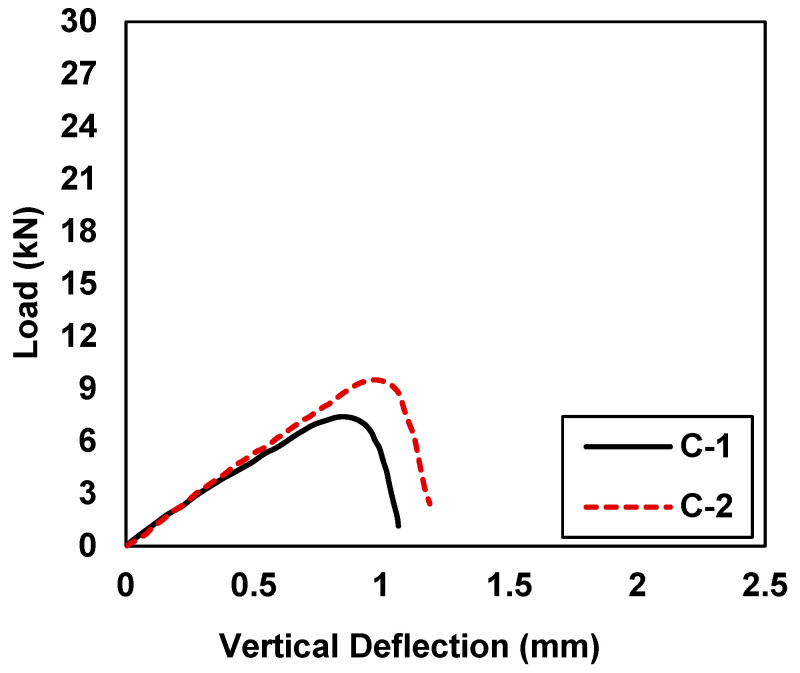
Load vs. midspan deflection for control samples (C-1 and C-2).

Figure 5 shows the load vs. vertical deflection values of the control samples C-1 and C-2. In Table 5, control sample C-1 shows maximum load and midspan deflection values of 7.40 kN and 0.850 mm, whereas the control sample C-2 shows maximum load and midspan deflection values of 9.50 kN and 0.970 mm. Both control samples behaved very similarly under flexure loading.

**Figure 6:**
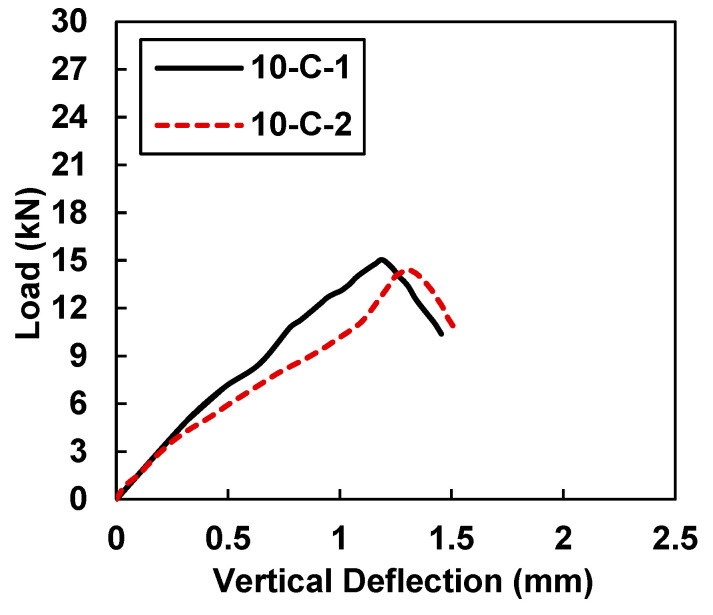
Load vs. midspan deflection for samples 10-C-1 and 10-C-2.

The specimen C-1 depicted peak load values lower than the C-2 specimen, which was mainly attributed to the heterogenous behavior of concrete and the sensitivity of un-reinforced concrete samples to tensile stresses. However, for comparison purposes, the average value of both specimens (C-1 and C-2) was used. Figure 6 shows the load-displacement behavior of 10-C-1 and 10-C-2, which were retrofitted with 10 mm CFRP strips. In comparison to the control samples, 10-C-1 and 10-C-2 showed improved behavior in terms of load-carrying capacity and deflection. Table 5 shows the peak load and midspan deflection values of 15.03 kN and 1.188 mm for sample 10-C-1, whereas sample 10-C-2 had maximum load and midspan deflection values of 14.43 kN and 1.308 mm, respectively. In comparison to the control samples (C-1 and C-2), on average an increase of 74.32% was observed in terms of the load-carrying capacity. Further, the deformation capacity was improved by 36.81% on average. A slight variation in the initial stiffness of sample 10-C-1 was observed. This variation might have been due to the uneven surface of sample 10-C-1 at the supports. Once the sample had settled itself on the support, it showed behavior similar to 10-C-2.

**Figure 7:**
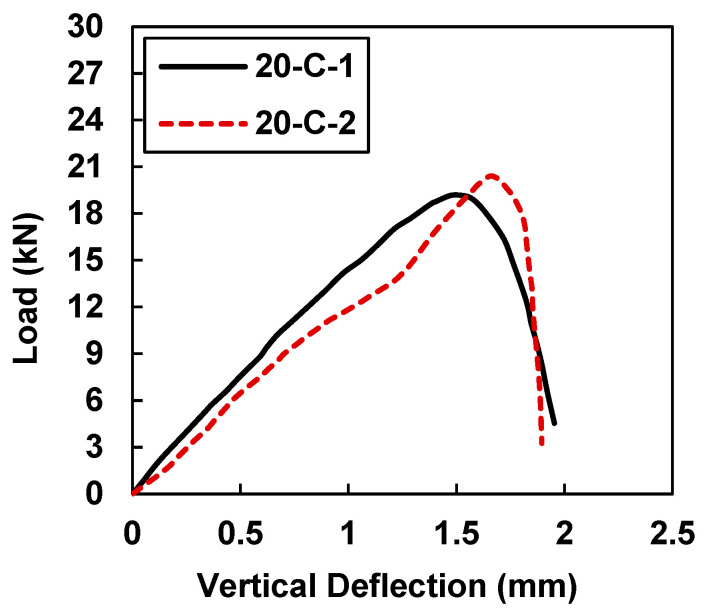
Load vs. midspan deflection for samples 20-C-1 and 20-C-2.

Figure 7 exhibits an average increase of 25% in the load-carrying capacity and a 35% increase in the vertical deflection for the specimens strengthened with the CFRP strips with widths equal to 20 mm.

By increasing the width of the CFRP strips from 10 mm to 20 mm, the beam samples were able to undergo large deflections, an indication of better ductile behavior. However, in the comparison to the control samples, an average increase of 132.19% was noted in the load-carrying capacity as well as an increase of 73.64% in the deflection. According to Table 5, the peak load and midspan deflection were 19.20 kN and 1.50 mm for sample 20-C-1, whereas sample 20-C-2 showed load and midspan deflection values of 20.04 kN and 1.667 mm.

**Figure 8:**
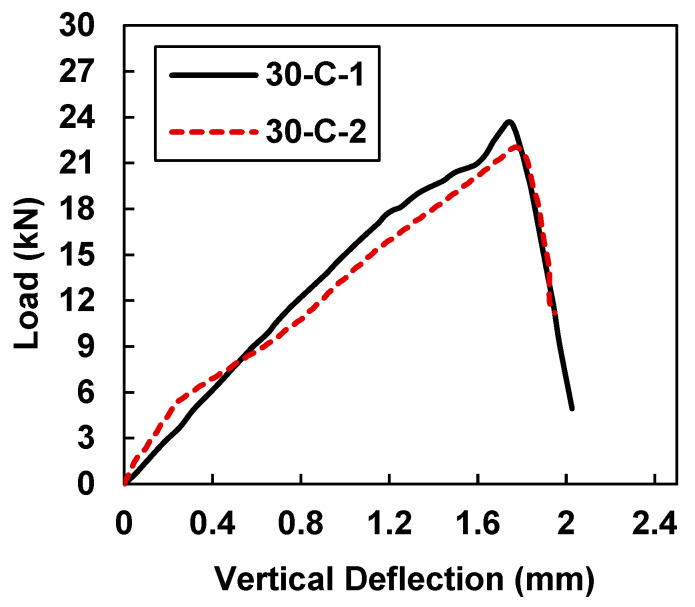
Load vs. midspan deflection for samples 30-C-1 and 30-C-2.

Figure 8 shows the maximum load and midspan deflection for samples 30-C-1 and 30-C-2.

In comparison to the control samples, on average increase of 152.54% in value was observed in the load-carrying capacity as well as a 92.31% increase in the deflection. Table 5 shows the maximum load and midspan deflection values of 23.66 kN and 1.734 mm for sample 30-C-1, whereas sample 30-C-2 showed maximum load and midspan deflection values of 22.04 kN and 1.776 mm. In comparison to the beam samples that were retrofitted with 20 mm CFRP strips, an average increase of 16.46% in the load-carrying capacity and a 10.76% increase in the deflection were observed. By increasing the width of the CFRP from 20 mm to 30 mm, a slight improvement in the displacement carrying capacity was observed.

**Figure 9:**
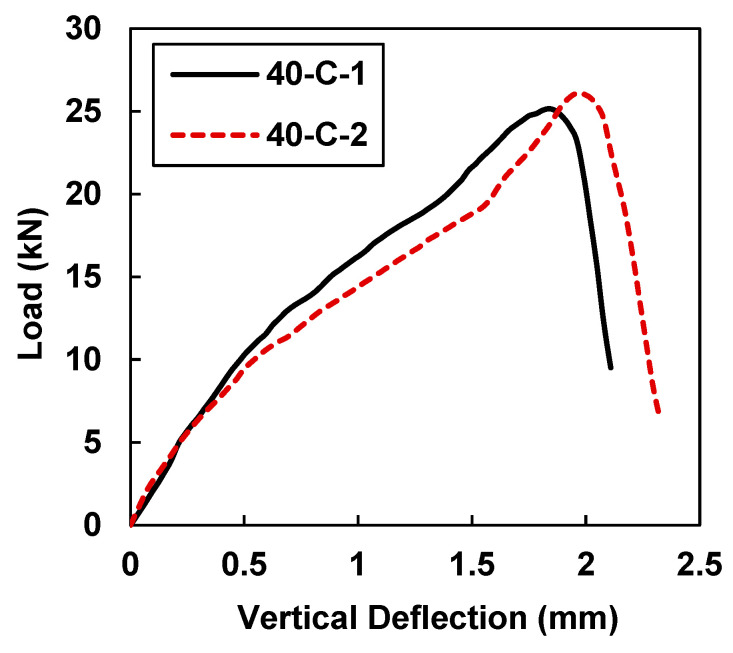
Load vs. midspan deflection for samples 40-C-1 and 40-C-2.

Figure 9 and Table 5 show the peak load and midspan deflection values of 25.16 KN and 1.841 mm for sample 40-C-1, whereas sample 40-C-2 had maximum load and midspan deflection values of 26.06 kN and 1.985 mm.

In comparison to the control samples, the load-carrying capacity and deflection increased by averages of 203% and 109.9%, respectively. However, in comparison with the specimens that were retrofitted with 30 mm CFRP strips, the peak load and vertical displacement of the 40-C specimens increased by average of 12% and 9.14%, respectively.

**Figure 10:**
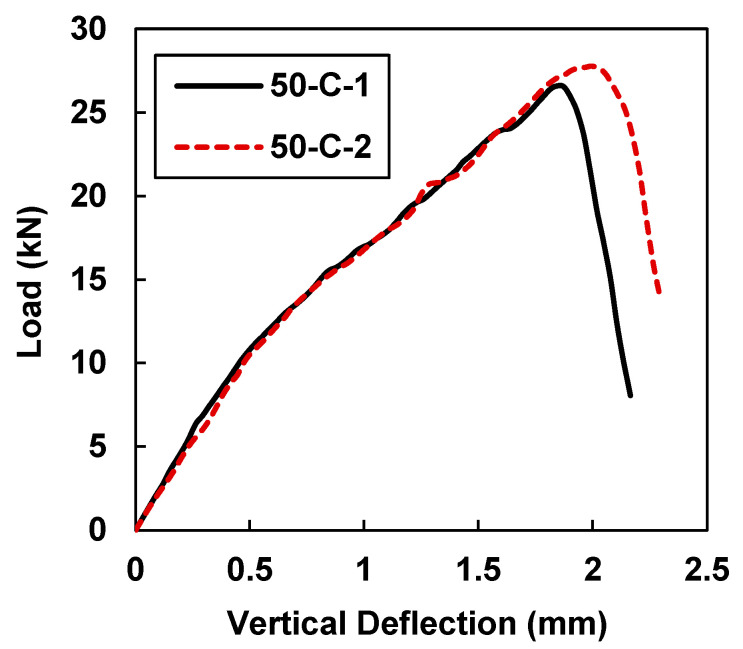
Load vs. midspan deflection for samples 50-C-1 and 50-C-2.

The load–deflection curves shown in Figure 10 for specimens 50-C-1 and 50-C-2 indicate the highest values of the peak load and midspan deflection in comparison to all other test specimens.

Table 5 shows that sample 50-C-1 had maximum load and midspan deflection values of 26.58 kN and 1.842 mm, while sample 50-C-2 had maximum load and midspan deflection values of 27.65 kN and 1.996 mm. In comparison to the beam samples that were retrofitted with 40 mm CFRP strips, there were average increases of 5.86% in load-carrying capacity and 0.52% in deflection. The deflection of the beam did not improve much by increasing the width of the CFRP strip from 40 mm to 50 mm. However, in comparison to the control samples, on average a 220% increase in value was observed in the load-carrying capacity, and a 111% increase was observed in the deflection.

**Figure 11:**
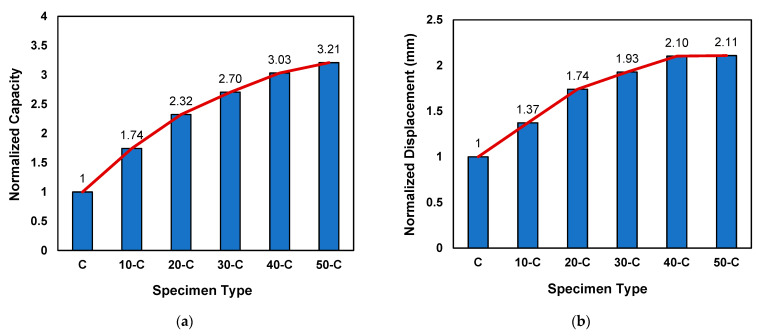
Effect of CFRP reinforcement on capacity and displacement: (**a**) normalized capacity and (**b**) normalized displacement.

In order to study the effect of CFRP reinforcement on the ultimate load-carrying capacity and deflection, the peak loads of all reinforced specimens (i.e., 10-C, 20-C, 30-C, 40-C, and 50-C) were normalized with reference to the control specimens (C) and are presented in Figure 11a,b, respectively.

Figure 11 illustrates a sharp increasing trend of peak load and deflection, with the increase in CFRP reinforcement until the width of 30 mm. However, the further increase in the CFRP reinforcement did not increase the capacity or displacement appreciably, which is quite evident for the cases of 40-C and 50-C in Figure 11, and in these cases the failure modes also changed from pure flexure failure to shear–flexure cracks that can be observed in Tables 7 and 8 in Section 7.3.

### 7.2. Peak Concrete Strain and CFRP Strain

Table 6 shows the experimental results summary of the samples in terms of peak concrete and CFRP strains and their average values. The peak values of the strains mentioned in Table 6 were measured at the instant when the sample reached its peak load-carrying capacity.

The specimen 50-C-2 showed the highest value of the strain in concrete and CFRP at the ultimate load. The debonding of CFRP started at the peak load, and strains corresponding to the peak loads can be categorized as debonding strain in all these cases. Due to good bond behavior and the uniform transfer of shear stresses between CFRP and concrete, the strain values of the concrete and CFRP for each tested specimen were found to be very close to each other until the sample reached its peak load.

**Figure 12:**
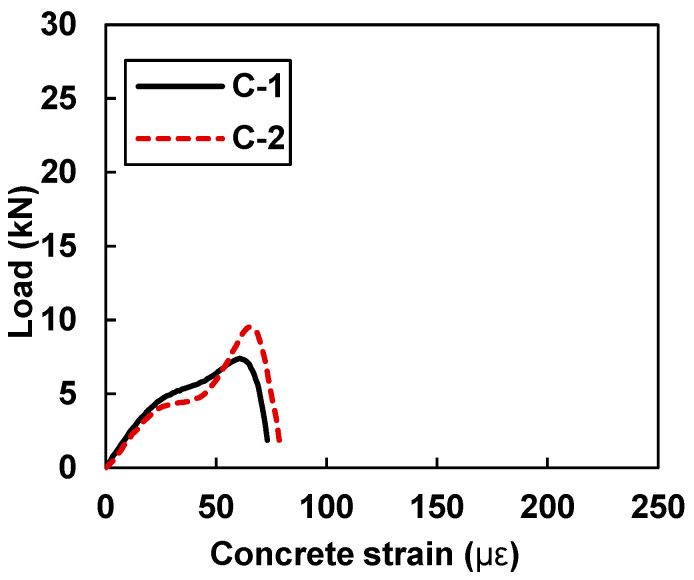
Load vs. concrete strain for samples C-1 and C-2.

Figure 12 shows the load versus concrete strain values of the control samples C-1 and C-2 without CFRP strips. In Table 6, control sample C-1 shows a peak concrete strain value of 60.49, whereas control sample C-2 shows a maximum concrete strain value of 65.14. The values of the peak concrete stains were monitored in the control sample at the instant when the sample reached its ultimate load-carrying capacity.

**Figure 13:**
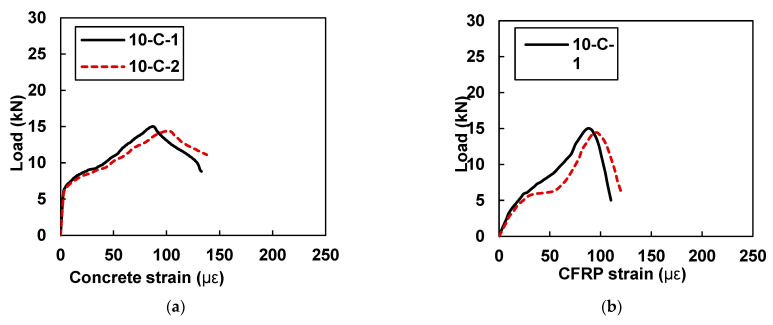
Load vs. strain curves for specimens with 10 mm CFRP strips (10-C-1 and 10-C-2): (**a**) strain in concrete and (**b**) concrete strain in CFRP.

Figure 13a,b shows the plots of load versus concrete microstrain and load versus CFRP microstrain for samples 10-C-1 and 10-C-2, respectively.

As reported in Table 6, specimen 10-C-1 showed a peak concrete strain value of 86.87 and a peak CFRP strain value of 88.14, whereas sample 10-C-2 showed a maximum concrete strain value of 95.97 and a peak CFRP strain value of 96.85. Close values of CFRP and concrete strain are evidence of good bonding behavior. In comparison to the control samples, the samples that were retrofitted with 10 mm CFRP plates exhibited good behavior in terms of deformation capacity, i.e., the average value of the concrete strain was found to be 21% higher than that of the concrete samples.

**Figure 14:**
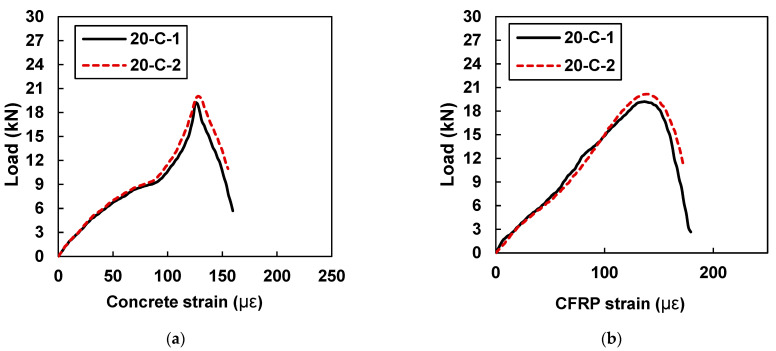
Load vs. strain curves for specimens with 20 mm CFRP strips (20-C-1 and 20-C-2): (**a**) strain in concrete and (**b**) concrete strain in CFRP.

Figure 14 shows plots of load versus concrete microstrain and CFRP microstrains for samples 20-C-1 and 20-C-2.

The strain values of the CFRP and concrete for both specimens were very close to each other, which is a sign of uniform shear stress transfer between the CFRP and concrete. In comparison to the beam samples retrofitted with 10 mm CFRP strips, an average increase of 36% in the value of the concrete and CFRP strain was noted. Specimen 20-C-1 showed a peak concrete strain value of 125.28 and a peak CFRP strain value of 132.40, whereas sample 20-C-2 showed a maximum concrete strain value of 127.70 and a peak CFRP strain value of 134.16. By increasing the width of the CFRP strip from 10 mm to 20 mm, the strain values were increased.

**Figure 15:**
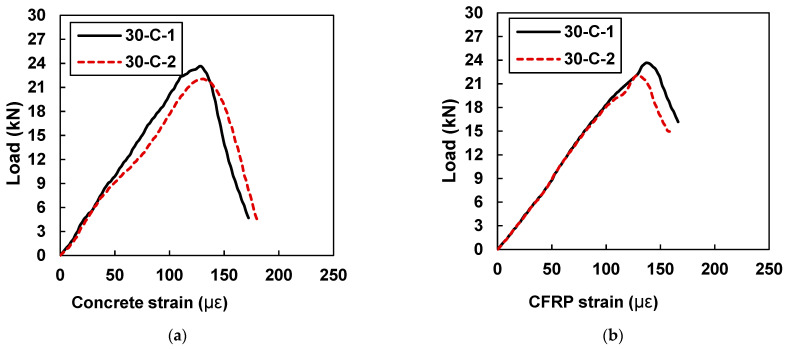
Load vs. strain curves for specimens with 30 mm CFRP strips (30-C-1 and 30-C-2): (**a**) strain in concrete and (**b**) concrete strain in CFRP.

Figure 15 shows the peak values of the concrete and CFRP strains for samples 30-C-1 and 30-C-2. Sample 30-C-1 showed a peak concrete strain value of 115 and a peak CFRP strain value of 128.37, whereas sample 30-C-2 showed a maximum concrete strain value of 203.95 and a peak CFRP strain value of 134.73. Table 6 indicates that the strain values only increased fractionally when the width of the CFRP plate increased from 20 mm to 30 mm, wight might have been due to weaker bond behavior between the CFRP and the concrete surface. However, with reference to the samples retrofitted with 10 mm plates, an average increase of 37% was noted in the values of concrete and CFRP strain.

**Figure 16:**
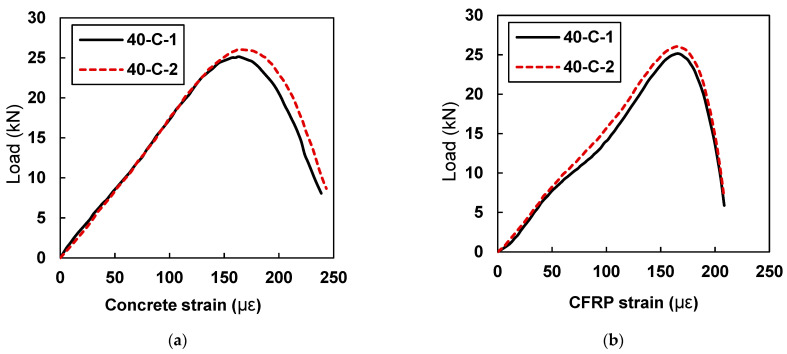
Load vs. strain curves for specimens with 40 mm CFRP strips (40-C-1 and 40-C-2): (**a**) strain in concrete and (**b**) concrete strain in CFRP.

In comparison to the beam sample retrofitted with a 30 mm CFRP plate, samples 40-C-1 and 40-C-2 showed (Figure 16) an average increase of 21% in strain values. Specimen 40-C-1 showed a peak concrete strain value of 163.20 and a peak CFRP strain value of 165.50, whereas sample 40-C-2 showed a maximum concrete strain value of 163.27 and s peak CFRP strain value of 165.62.

**Figure 17:**
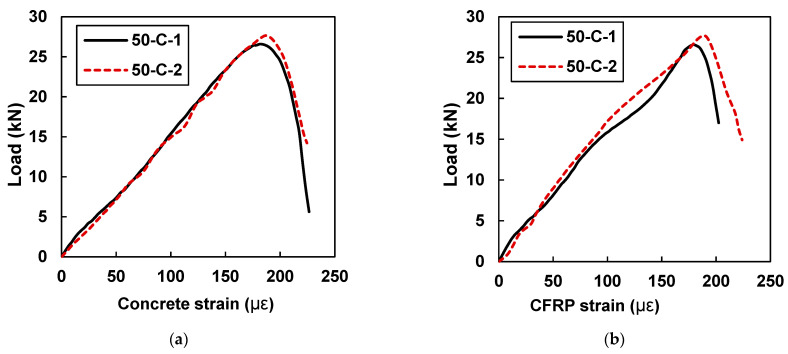
Load vs. strain curves for specimens with 50 mm CFRP strips (50-C-1 and 50-C-2): (**a**) strain in concrete and (**b**) concrete strain in CFRP.

Figure 17 exhibits that, in comparison to all tested beam samples, 50-C-1 and 50-C-2 showed the highest values of concrete and CFRP strains. In comparison to the beam samples retrofitted with 40 mm CFRP strips, an average increase of 11% was found in the concrete and CFRP strain values.

### 7.3. Failure Modes of Beam Samples

The initial cracking pattern and the final overall failure modes of all specimens tested against the flexure loading were captured and are summarized in Table 7 and Table 8, respectively.

Due to the brittle behavior, sudden failure without initial warning occurred in the control specimens (C-1 and C-2), and the failure mode was regarded as pure flexural. It is quite evident in Table 7 for the case of control specimen (C) that the initial flexural crack was formed at the bottom surface near the midspan of the beam, and it then propagated vertically to the top. For the specimens retrofitted with 10 mm, 20 mm, and 30 mm CFRP strips (i.e., the 10-C, 20-C, and 30-C cases in Table 7 and Table 8), it was observed that the failure initiated with the debonding of the CFRP strip from the bottom of the concrete beam sample, followed by a flexural crack near the midspan of the beam.

For samples that were strengthened with a CFRP strip with a width of 30 mm or less, no redistribution of the stresses was observed. This was due to the dominance of the concrete’s tensile cracking behavior. The provided CFRP reinforcement was not adequate to control the tensile cracking of the concrete at the peak load level, and debonding started, which could be witnessed with vertical flexural cracking in all these samples. However, in the case of the 40-C and 50-C samples, CFRP provided sufficient restraint against the pure flexural tensile cracking, and concrete shear stresses came into action, which resulted in the redistribution of stresses from pure flexure to flexure–shear behavior, and ultimately the samples failed in the flexural shear mode. It is difficult to relate this phenomenon with the moment redistribution of the reinforced concrete beams, as the failure pattern and collapse mechanism largely depend on the moment redistribution and the formation of plastic hinges. Similarly, a study conducted by Guella, F and Baji, H also discussed a similar behavior in relation to the moment redistribution in concrete structures [53,54].

In the case of the 40-C and 50-C samples, the failure modes shifted from pure flexure to shear flexural, as shown in Table 7 and Table 8. For 40-C and 50-C, the failure initiated with the debonding of the CFRP strip from the concrete surface, followed by an inclined crack at a 45-degree angle. In all test specimens, the final failure at the end of the testing was due to the separation of the CFRP strips from the bottom surface of the beam sample, which can be observed in Table 8. In all CFRP-strengthened samples, the failure mainly happened in the concrete. The CFRP strips remained undamaged and did not show any signs of distress or cracking. This was due to the significantly higher stiffness of CFRP compared to concrete, which was deliberately employed to study the debonding strain of the CFRP from the concrete.

## 8. Conclusions

The influence of different widths of CFRP on the debonding strain and failure patterns of concrete prims were investigated in this research paper. The CFRP strip widths were varied gradually to see their effect on peak loads, debonding strain, and deformation capacity. Based on the experimental test data, analysis, and discussion, the following conclusions have been drawn:

- CFRP improved the load-carrying capacity and deformation capacity of the retrofitted beam samples. However, this increase was not directly proportional to increase in the cross-sectional area of the CFRP. An effective substrate (epoxy, concrete, and interface) plays a significant role in strength gain.
- An improvement in the peak strain values of concrete and CFRP was observed with an increase in the width of the CFRP. The maximum values of the strain (concrete and CFRP) were observed in beam samples retrofitted with 50 mm CFRP strips. The peak strain achieved in CFRP depends upon the effective substrate area (concrete, epoxy, and its interface). By increasing the width of the CFRP, the effective contribution of the substrate (concrete, epoxy, and its interface) increased, which assisted CFRP in achieving better strain. However, this peak strain was significantly lower than the ultimate strain of the CFRP and concrete. For samples 10-C, 20-C, and 30-C, failure was initiated due to the flexure rupture of concrete across the entire cross section, followed by debonding failure, whereas for 40-C and 50-C flexure–shear failure (diagonal cracks) was observed, which was followed by the debonding of the CFRP strip. Due to the wider strips, the effective substrate also became wider, which assisted the CFRP in gaining better strain values compared to 10-C, 20-C, and 30-C.
- The transfer of concrete and CFRP strains is highly dependent on good bonding behavior between these two materials. No damage to CFRP strips was observed, which shows that the CFRP used was significantly stronger. It is good for strength gain but could be costly if other system strength parameters such as the epoxy and concrete are weaker. In order to achieve an efficient strengthening design, the concrete surface properties shall be thoroughly assessed for effective strain transfers to FRP.
- Specimens strengthened with 40 mm and 50 mm CFRP strips had flexural shear failure. This implies that increasing the CFRP reinforcement beyond this point will not improve the beam’s overall load-carrying capacity until it is strengthened or reinforced for shear forces and stresses.

## Figures and Tables

**Table 1:** Designation of test specimens.

Sr. No.	Sample Designation	Width of CFRP Strip (mm)
1	C-1	N/A
2	C-2	
3	10-C-1	10
4	10-C-2	10
5	20-C-1	20
6	20-C-2	20
7	30-C-1	30
8	30-C-2	30
9	40-C-1	40
10	40-C-2	40
11	50-C-1	50
12	50-C-2	50

**Table 2:** Properties of concrete [4].

Material Property	Value
Compressive strength of the concrete, *f_c_′*	32 MPa
Modulus of elasticity of the concrete, *E_c_*	26.59 GPa
Poison’s ratio of the concrete, *υ*_c_	0.18
Coefficient of thermal expansion of the concrete, α_c_	10 × 10^−6^/°C
Shear modulus of the concrete, *G_c_*	10.63 GPa ^†^

**^†^** as per ACI 318-19 (51) commentary of section 6.6.3.1, the shear modulus can be taken as 0.4 *E_c_*.

**Table 3:** Properties of CFRP [4].

Specific Gravity	Tensile Strength	Tensile Modulus	Bending Strength	Bending Modulus	Coefficient of Thermal Expansion	Ultimate Elongation
	**(MPa)**	**(GPa)**	**(MPa)**	**(GPa)**	**(10^−6^/°C)**	**(%)**
1.5	1600	120	104	72	0.2	1.8

**Table 4:** Properties of the epoxy [4].

Specific Gravity	Tensile Strength	Tensile Shear Bond Strength	Bending Strength	Compressive Elasticity Modulus
	**(MPa)**	**(MPa)**	**(MPa)**	**(GPa)**
1.4	20	9.6	45	1.5

**Table 5:** Experimental results summary of peak load and mid-span deflection.

Sr. No.	Sample Designation	Peak Load	Average Peak Load	Midspan Deflection	Average Midspan Deflection	Normalized Deflection
		**(kN)**	**(kN)**	**(mm)**	(mm)	-
1	C-1	7.40	8.45	0.850	0.91	1
2	C-2	9.50		0.970		
3	10-C-1	15.03	14.73	1.188	1.248	1.371
4	10-C-2	14.43		1.308		
5	20-C-1	19.20	19.62	1.500	1.58	1.735
6	20-C-2	20.04		1.667		
7	30-C-1	23.66	22.85	1.734	1.75	1.736
8	30-C-2	22.04		1.776		
9	40-C-1	25.16	25.61	1.841	1.91	2.098
10	40-C-2	26.06		1.985		
11	50-C-1	26.58	27.11	1.842	1.92	2.01
12	50-C-2	27.65		1.996		

**Table 6:** Experimental results summary of strain in concrete and CFRP.

Sr. No	Sample Designation	Peak Load	Strain in Concrete	Average Strain in Concrete	Strain in CFRP	Average Strain in CFRP
		**(kN)**	**(*µ*mm/mm)**	**(*µ*mm/mm)**	**(*µ*mm/mm)**	**(*µ*mm/mm)**
1	C-1	7.40	60.49	62.82	N/A	N/A
2	C-2	9.50	65.14			
3	10-C-1	15.03	86.87	91.42	88.14	92.49
4	10-C-2	14.43	95.97		96.85	
5	20-C-1	19.20	125.28	126.49	132.40	133.28
6	20-C-2	20.04	127.70		134.16	
7	30-C-1	23.66	128.37	128.96	134.73	132.70
8	30-C-2	22.04	129.55		130.67	
9	40-C-1	25.16	163.20	163.24	165.50	165.56
10	40-C-2	26.06	163.27		165.62	
11	50-C-1	26.58	181.20	183.88	179.16	183.41
12	50-C-2	27.65	186.55		187.65	

**Table 7:** Failure pattern of the tested beam samples.

Specimen	Failure Type	Failed Specimen
Control C	Pure Flexure	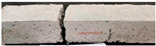
10-C	Pure Flexure	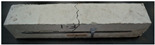
20-C	Pure Flexure	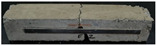
30-C	Pure Flexure	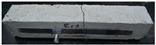
40-C	Flexural Shear	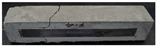
50-C	Flexural Shear	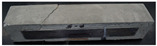

**Table 8:** Flexure and shear crack propagation of the tested beam samples.

Specimen	Failure Type	Failed Specimen
10-C	Pure Flexure	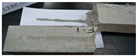
20-C	Pure Flexure	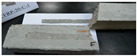
30-C	Pure Flexure	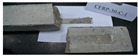
40-C	Flexural Shear	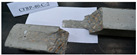
50-C	Flexural Shear	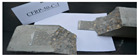

## Data Availability

Not applicable.
